# Electrically Induced Calcium Handling in Cardiac Progenitor Cells

**DOI:** 10.1155/2016/8917380

**Published:** 2016-10-12

**Authors:** Joshua T. Maxwell, Mary B. Wagner, Michael E. Davis

**Affiliations:** ^1^Wallace H. Coulter Department of Biomedical Engineering, Emory University School of Medicine, Atlanta, GA, USA; ^2^Division of Pediatric Cardiology, Department of Pediatrics, Emory University School of Medicine, Atlanta, GA, USA; ^3^Children's Heart Research and Outcomes (HeRO) Center, Children's Healthcare of Atlanta and Emory University, Atlanta, GA, USA

## Abstract

For nearly a century, the heart was viewed as a terminally differentiated organ until the discovery of a resident population of cardiac stem cells known as cardiac progenitor cells (CPCs). It has been shown that the regenerative capacity of CPCs can be enhanced by* ex vivo* modification. Preconditioning CPCs could provide drastic improvements in cardiac structure and function; however, a systematic approach to determining a mechanistic basis for these modifications founded on the physiology of CPCs is lacking. We have identified a novel property of CPCs to respond to electrical stimulation by initiating intracellular Ca^2+^ oscillations. We used confocal microscopy and intracellular calcium imaging to determine the spatiotemporal properties of the Ca^2+^ signal and the key proteins involved in this process using pharmacological inhibition and confocal Ca^2+^ imaging. Our results provide valuable insights into mechanisms to enhance the therapeutic potential in stem cells and further our understanding of human CPC physiology.

## 1. Introduction

The heart was considered a postmitotic organ incapable of regeneration until the discovery of a resident population of cardiac stems cells brought about the potential for cardiac tissue regeneration [1]. Known as cardiac progenitor cells (CPCs) and primarily found in the myocardium, these cells have cardiogenic gene expression as well as the stem cell marker c-kit [2]. Since the first reports of c-kit^+^ CPCs, there has been controversy surrounding the ability of these cells to replace cardiac myocytes [1, 3–7]. Despite earlier reports that CPCs could replace damaged myocardium [1, 8], recent lineage tracking studies have provided compelling evidence that these cells do not become cardiac myocytes* in vivo* [4, 5, 7]. However, it has been shown that the regenerative capacity of these cells can potentially be enhanced by* ex vivo* modification. Several laboratories have demonstrated the feasibility and utility of* ex vivo* manipulation of adult stem cells modified by genetic engineering [9–13] or exposure to environmental, chemical, and biological treatments prior to delivery [9, 14–17]. The idea behind these strategies is to isolate a patient's CPCs and expand and modify them to create a more therapeutic phenotype.

Electrical stimulation is one treatment known to enhance cardiogenic potential of various stem cells via activation of calcium (Ca^2+^) signaling in adult cells [18–22]. Ca^2+^ is an integral second messenger in the heart, regulating the processes of excitation-contraction coupling (action potential-mediated Ca^2+^ entry triggers contraction) and excitation-transcription coupling (action potential-mediated Ca^2+^ entry triggers changes in gene expression). Previous reports showed that electrical stimulation of adult cardiac adipose tissue-derived progenitor cells caused changes in cell phenotype and genetic machinery, making them more suitable for cardiac regenerative approaches [19]. Furthermore, electrical stimulation of adult progenitor cells has been shown to induce a variety of responses, such as cytoskeletal rearrangements, migration, proliferation, and differentiation, as well as the* de novo* expression of the late cardiac sarcomeric proteins, troponin T and cardiac alpha actinin, and increase the expression of connexin 43 and its relocation to the cell membrane [19–22]. Recent data indicate that Ca^2+^ is involved in assembly of contractile apparatus and localization of key cardiogenic transcription factors, such as myocyte enhancer factor 2c, in CPCs [23]. Despite these results, the mechanisms behind the enhanced cardiospecific gene expression and the increased therapeutic potential of adult CPCs reported with electrical stimulation have not been elucidated [23–25]. Furthermore, these experiments have been conducted with very little knowledge of the basic composition of these cells to direct their efforts. Systematic studies of the regulation of Ca^2+^ handling in CPCs would substantially advance our knowledge of CPCs physiology and their potential in cardiac repair, providing fundamental information that could be used for developing new therapeutic approaches to improve myocardial repair and regeneration.

Toward that end, we aimed to characterize the mechanism of electrically induced Ca^2+^ handling in human c-kit^+^ cardiac progenitor cells. In this study, we have identified a novel property of CPCs to respond to electrical stimulation by initiating intracellular Ca^2+^ oscillations. CPCs were isolated from human atrial tissue and pooled from multiple donors to prevent patient-specific results in our experiments. These cells were loaded with the Ca^2+^-sensitive dye fluo-4/AM to measure the cytosolic and nuclear Ca^2+^ oscillations in response to acute electrical stimulation using live cell confocal microscopy. We characterized the Ca^2+^ signal by measuring the amplitude of cytosolic and nuclear Ca^2+^ oscillations elicited by electrical stimulation, identified the mechanism by which Ca^2+^ oscillations initiate and propagate throughout the cells, and also determined the key proteins involved in this process. This project may have implications on many stem cell therapies and provide us with valuable insights into mechanisms to enhance the therapeutic potential of stem cells and further our understanding of human CPC physiology.

## 2. Materials and Methods

### 2.1. Patient Aspects of the Proposed Research

Human cardiac progenitor cells (CPCs) used in this study were obtained under IRB approval which allows for isolation of CPCs from deidentified atrial waste tissue removed during surgical repair of congenital heart defects. Patients were not subject to any additional risks by involvement in this study. Confidentiality of patient information was assured as we did not record the patients' names or demographic information and did not contact the patients. A clinical research nurse obtained needed information such as age, gender, ethnicity, diagnosis, and drug therapy, but tissue samples were deidentified and data obtained from the biopsy were identified by an internal lab number only. No preference as to gender, race, or ethnicity was used for patient enrollment.

### 2.2. Reagents

All chemicals and reagents were purchased from Sigma-Aldrich (St. Louis, MO, USA), unless noted otherwise. Stock solutions of tetracaine and 2-aminoethoxydiphenyl borate (2-APB) were made in methanol and ethanol, respectively, and diluted to work concentrations in Tyrode's solution. The vehicle was added to Tyrode's solution for control experiments. Primary antibodies for the L-type Ca^2+^ channel and the inositol 1,4,5-trisphosphate receptor were obtained from Santa Cruz Biotechnology (Dallas, TX, USA), and primary antibody for the ryanodine receptor and secondary antibodies Alexa Fluor 488 and Alexa Fluor 647 were purchased from Thermo Fisher Scientific (Waltham, MA, USA).

### 2.3. Culturing of Human Cardiac Progenitor Cells

Human child (aged 1–5 years) cardiac progenitor cells were isolated from biopsied tissue using c-kit antibody-conjugated magnetic beads and propagated in culture as previously described [26]. Briefly, tissue was minced and digested with type 2 collagenase (1 mg/mL) in Hank's balanced salt solution and passed through a 70 *μ*m filter. Cells were incubated with Dynabeads (Thermo Fisher Scientific, Waltham, MA, USA) conjugated to a c-kit antibody (Santa Cruz Biotechnology, Dallas, TX, USA) prior to magnetic sorting. Sorted cells were plated in a T-75 tissue culture flask and expanded to confluence. Cells were pooled from 4–6 donors to reduce variability. CPC culture media consisted of Ham's F-12 basal media (Mediatech, Manassas, VA, USA) along with 10% fetal bovine serum (Atlanta Biologicals, Flowery Branch, GA, USA), 1% penicillin, 1% streptavidin, 1% L-glutamine, and 0.1 *μ*g/mL basic fibroblast growth factor. Prior to experiments, cells were plated on glass coverslips and allowed to attach overnight at 37°C.

### 2.4. Intracellular Calcium Measurements

Cells were loaded with 10 *μ*M fluo-4/AM for 20 min followed by a 20 min wash in Tyrode's solution for deesterification of the dye. Electrical field stimulation was applied using an IonOptix MyoPacer Cell Stimulator (10 ms duration, 32 V; Westwood, MA, USA) and fluo-4 was excited at 488 nm and emission collected at 515 ± 15 nm using laser scanning confocal microscopy (FV1000, Olympus, Melville, NY, USA). Calcium oscillation measurements were acquired from CPCs during 0.5 or 1 Hz stimulation bathed in Tyrode's solution, which contained (in mM) 130 NaCl, 4 KCl, 2 CaCl_2_, 1 MgCl_2_, 10 D-glucose, and 10 Hepes, pH 7.4 with NaOH. All experiments were performed at room temperature (20–24°C). Data were analyzed using Olympus FV1000 FluoView software and Fiji [27].

### 2.5. Membrane Staining

Cell membranes and transverse tubular structures were visualized with the membrane-bound fluorescent probe Di-8-ANEPPS (Thermo Fisher Scientific, Waltham, MA, USA) by 2-dimensional confocal microscopy. Cells were loaded for 15 min with Di-8-ANEPPS (5 *μ*M) in Tyrode's solution and the indicator was excited at 488 nm, and emission was measured at >600 nm.

### 2.6. Immunocytochemistry

CPCs were plated on glass coverslips, fixed in 4% paraformaldehyde, permeabilized with 0.1% Triton, and blocked with 3% BSA/PBS for 2 hrs. Cells were treated with the appropriate primary (Cav2.1, type 2 IP_3_R, and RyR2) and secondary antibodies for 2 hrs at dilution of 1 : 500. After washing with PBS, VECTASHIELD HardSet Antifade Mounting Medium containing DAPI (Vector Laboratories, Burlingame, CA, USA) was used to mount the coverslips onto glass slides for visualization.

### 2.7. Long-Term Electrical Stimulation

CPCs were seeded at a density of 4 × 10^5^ cells/well in a 6-well C-dish (IonOptix, Westwood, MA, USA) and incubated in stimulation media. CPC stimulation media consisted of Ham's F-12 basal media along with 10% fetal bovine serum, 1% penicillin, 1% streptavidin, 1% L-glutamine, and 2 mM CaCl_2_. The C-Pace EP Culture Pacer (IonOptix, Westwood, MA, USA) was used for culture stimulation at 0.5 Hz, 10 ms duration, 32 V for 72 hrs. Media were changed every 24 hrs.

### 2.8. Cell Alignment Quantification

After 72 hours of electrical stimulation, cells were immediately fixed in 4% paraformaldehyde for 20 minutes at room temperature. Unstimulated cells cultured in stimulation media for 72 hrs or cells stimulated in Ca^2+^-free media for 72 hrs were also processed and stained as described below for basal and Ca^2+^-dependent alignment quantification. Following three washes in 1x PBS, the cells were permeabilized with 0.1% Triton in 1x PBS. Cells were again washed and then blocked in 3% bovine serum albumin for 1 hour at room temperature. Cells were then stained with 10 *μ*g/mL fluorescein-5-maleimide for 1 hour at room temperature in the absence of light, followed by washing. Cells were washed prior to imaging on an Olympus IX70 inverted fluorescent microscope (Melville, NY, USA). Two-dimensional fluorescent images were acquired using the 20x objective and analyzed with Fiji software using the directionality function to obtain the primary angle of the cells and the percent of cells within one standard deviation of the direction of the applied electrical field [27].

### 2.9. Data Presentation

Fluorescence traces and confocal line scan data are presented as individual observations representative of multiple recordings or as the average of multiple recordings. Fluorescence traces were background subtracted and plotted as *F*/*F*_0_, where *F*_0_ is the basal fluorescence in resting cells prior to electrical stimulation (for initial oscillation) or the diastolic fluorescence just before an oscillation (for subsequent oscillations). Fluorescent cellular images are presented as two-dimensional images representative of multiple trials. Summary data are presented as the mean ± SD of *n* measurements, where *n* is the total number of cells from 4 different pools of cells. Statistical comparisons between groups were performed with Student's *t*-test. Differences were considered statistically significant at *p* < 0.05.

## 3. Results and Discussion

### 3.1. Global Ca^2+^ Oscillations in Electrically Stimulated Human Cardiac Progenitor Cells

**Figure 1:**
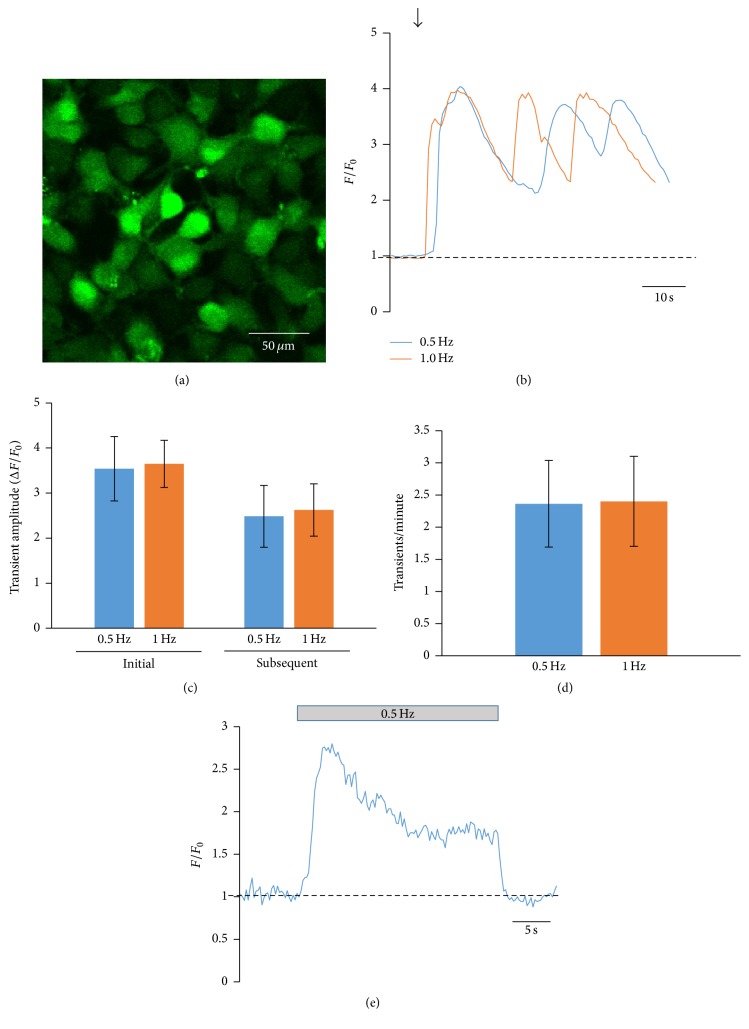
Whole-cell Ca^2+^ oscillations in electrically stimulated CPCs. (a) 2D fluorescent image of child CPCs loaded with fluo-4/AM. Fluo-4/AM-loaded hcCPCs were electrically stimulated at 0.5 Hz (blue) or 1 Hz (orange). Representative recordings of Ca^2+^ oscillations are shown in (b). The arrow indicates the start of electrical stimulation. The amplitude (Δ*F*/*F*_0_) and frequency (oscillations/minute) of the oscillations are summarized in (c) and (d), respectively. (e) Representative recording of cytosolic Ca^2+^ before, during, and after 0.5 Hz electrical stimulation. The bar above the trace shows the time during the recording when electrical stimulation was applied. Data in (e) represent mean ± SD. No significance was found by Student's *t*-test. *n* = 14 cells from 4 different pools of cells for all measurements.

We have obtained human cardiac tissue from children undergoing repair for congenital heart defects and utilized these samples for isolation of human c-kit^+^ CPCs by a method previously described by our laboratory [26, 28–31]. Our technique results in a population of cells that are ~94% positive for the stem cell marker c-kit [26]. CPCs were loaded with the Ca^2+^ dye fluo-4/AM and stimulated at 0.5 or 1 Hz.  Figure 1 shows representative Ca^2+^ oscillations in CPCs elicited by electrical stimulation. A rapid rise in cytosolic Ca^2+^ was observed followed by frequency-independent cytosolic Ca^2+^ oscillations (Figure 1(b)). After the initial oscillation, subsequent oscillations did not decline back to baseline levels but instead came to a new level of cytosolic “diastolic” Ca^2+^ presumably due to loading of the cell with Ca^2+^. We then quantified the amplitude of these Ca^2+^ oscillations and compared them based on the frequency of electrical stimulation (0.5 or 1 Hz). Quantification of the amplitude of the Ca^2+^ oscillations showed no significant difference between the initial oscillation amplitudes at 0.5 or 1 Hz or the subsequent oscillation amplitudes (Figure 1(c)). Furthermore, the stimulation frequency did not affect the rate at which Ca^2+^ oscillations were observed in these cells (Figure 1(d)). Despite the subsequent Ca^2+^ oscillations not being dependent on the stimulation frequency, it is clear that electrical stimulation initiates the Ca^2+^ oscillations and also plays a part in maintaining cytosolic Ca^2+^ in these cells, as cessation of electrical stimulation caused a decrease in cytosolic Ca^2+^ back to baseline levels and abolished the Ca^2+^ oscillations (Figure 1(e)). Additionally, all cells recorded displayed an increase in cytosolic Ca^2+^ in response to electrical stimulation. We did not observe spontaneous Ca^2+^ oscillations in these cells under our recording conditions as has been previously reported, and the amplitude of our electrically induced oscillations is approximately 4–6 times greater than that reported for spontaneous Ca^2+^ oscillations [32].

**Figure 2:**
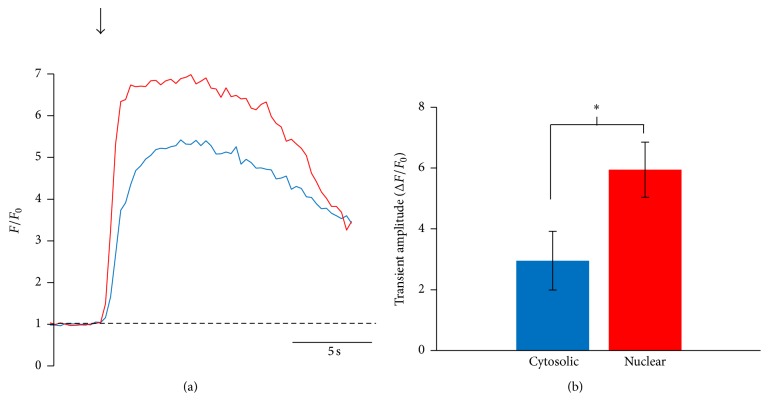
Nuclear Ca^2+^ increases more than cytosolic Ca^2+^ in response to electrical stimulation. Fluo-4/AM-loaded hcCPCs were stimulated at 0.5 Hz and the amplitudes of the nuclear and cytosolic Ca^2+^ oscillations were analyzed. Representative traces of nuclear (red) and cytosolic (blue) Ca^2+^ oscillations are shown in (a), and amplitudes are summarized in (b). Data represent mean ± SD. ^*∗*^ *p* < 0.05; Student's *t*-test. *n* = 16 cells from 4 different pools of cells for all points.

The amplitude of nuclear Ca^2+^ oscillations was also measured in electrically stimulated CPCs and these oscillations were of significantly higher amplitude than the corresponding cytosolic Ca^2+^ oscillations.  Figure 2(a) shows representative traces of nuclear and cytosolic Ca^2+^ oscillations taken from the same cell and aligned in time. These data are summarized in Figure 2(b). Our finding that nuclear Ca^2+^ oscillations were of significantly higher amplitude than cytosolic Ca^2+^ oscillations may indicate a possible role for the increased nuclear Ca^2+^ in regulation of Ca^2+^-dependent proteins and transcription factors leading to differentiation and lineage commitment of CPCs. Activation of Ca^2+^ signaling by electrical stimulation has been shown to cause changes in the phenotype and genotype in multiple types of adult progenitor cells [19–22]. We did not observe spontaneous Ca^2+^ release events (Ca^2+^ waves or Ca^2+^ sparks) in unstimulated cells under our experimental conditions nor did the imaging laser activate Ca^2+^ release events. Together, these data show that electrical stimulation of CPCs elicits a robust cytosolic and nuclear Ca^2+^ response from these cells.

### 3.2. Ca^2+^ Oscillations Propagate in a Wave-Like Fashion in CPCs

**Figure 3:**
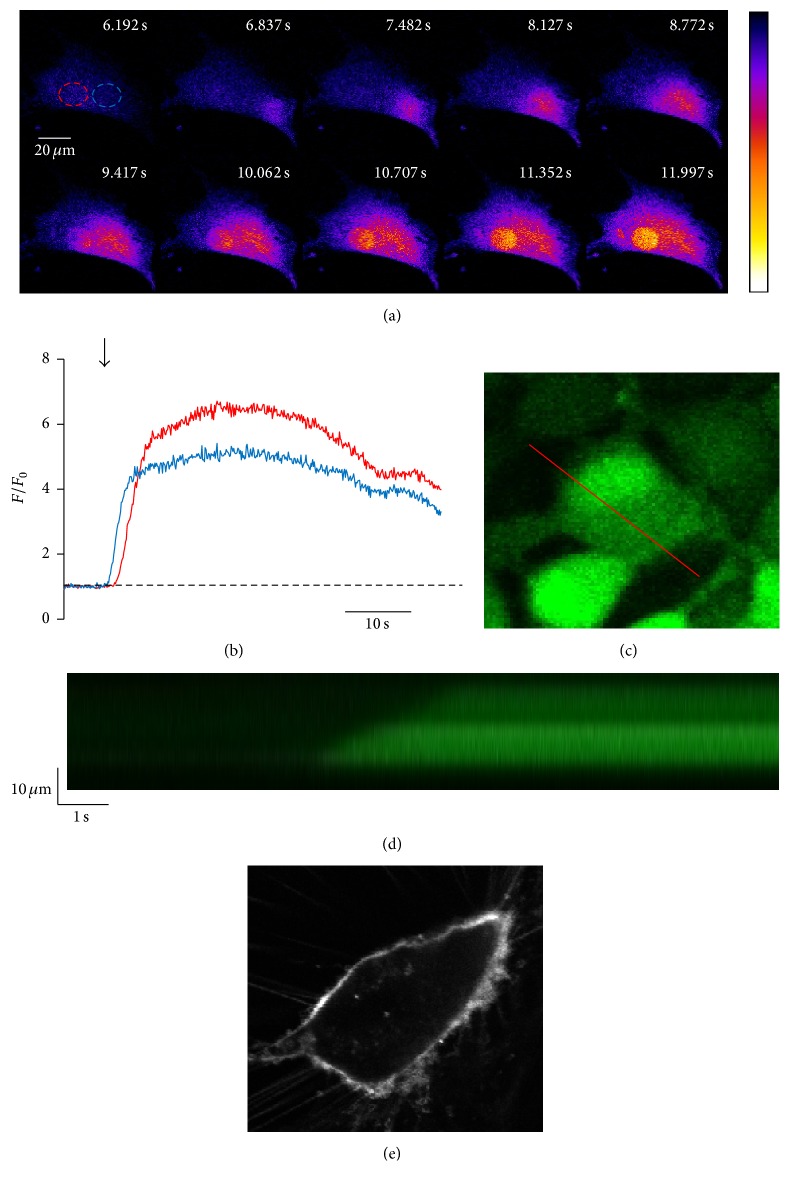
Ca^2+^ oscillations in electrically stimulated CPCs show spatial heterogeneity and propagate in a wave-like fashion. ((a) and (b)) Representative 2D high-speed confocal image montage of the activation of a Ca^2+^ oscillation in a CPC loaded with fluo-4/AM. ROIs (red: nuclear, blue: cytosolic) indicated in (a) are graphed in (b). ((c) and (d)) Measured from the red line shown in (c), the confocal line scan in (d) shows that initiation of Ca^2+^ release at one edge of the cell propagates through the cell to the other side. (e) Representative image of a CPC stained with Di-8-ANEPPS.

The Ca^2+^ oscillations were further analyzed by two-dimensional and high-speed line scan confocal imaging to determine the spatiotemporal properties of Ca^2+^ oscillations in CPCs.  Figure 3(a) shows a representative 2D high-speed confocal image montage of the activation of a Ca^2+^ oscillation in a CPC. Interestingly, Ca^2+^ release begins in a focal region in the periphery of the cell and then propagates in a wave-like fashion through the cytosol and the nucleus. Fluorescent traces from a cytosolic region (blue) and the nucleus (red) are shown in Figure 3(b). As shown in the traces, the cytosolic Ca^2+^ in the region near the site of Ca^2+^ release activation increases first (blue trace), and the Ca^2+^ signal propagates outward through the cytosol and eventually reaches the nucleus and an increase in the nuclear Ca^2+^ is seen (red trace). Again, a larger nuclear Ca^2+^ oscillation compared to the cytosolic Ca^2+^ oscillation can be seen in these traces, with the red nuclear trace reaching a higher amplitude than that of the blue cytosolic trace. These traces show the spatiotemporal heterogeneity of Ca^2+^ release activated by electrical stimulation in CPCs and show that the Ca^2+^ oscillations in these cells propagate in a wave-like fashion, initiating at the periphery of the cell and propagating through the cytosol and nucleus.

Confocal line scans were used to confirm our 2D data described above.  Figure 3(c) shows the placement of the line for recording, and Figure 3(d) is the resulting line scan. This scan is representative of 12 trials. As shown, the Ca^2+^ release activated by electrical stimulation starts at the periphery of the cell and propagates across the line in a wave-like fashion over time. We also looked for cell membrane invaginations that may identify preferential sites in the CPC similar to transverse tubules (T-tubules) in adult ventricular myocytes. T-tubules are invaginations of the sarcolemmal membrane in cardiac myocytes that bring the surface membrane in close proximity to the sarcoplasmic reticulum (SR) membrane allowing for efficient coupling of Ca^2+^ entry through sarcolemmal L-type Ca^2+^ channels and activation of intracellular Ca^2+^ release by ryanodine receptors on the SR [33]. Staining of CPCs with the membrane dye Di-8-ANEPPS did not reveal any invaginations of the surface membrane indicating that this feature did not contribute to Ca^2+^ release activation in CPCs (Figure 3(e)). In summary, the electrically induced Ca^2+^ release in CPCs very much resembles Ca^2+^ wave propagation in adult ventricular myocytes [34]. Despite their activation in the general periphery of the cell, they do not appear to be activated in any more localized preferential sites within the cell or at the same region in the periphery in subsequent Ca^2+^ oscillations. Consistent with the finding that there were no preferential sites of Ca^2+^ release during electrical stimulation, we did not find evidence of T-tubules which have been shown to be preferential release sites in ventricular myocytes [33]. We hypothesize that Ca^2+^ release initiation occurs at the periphery of the cell where the cell membrane and endoplasmic reticulum may be closest together to facilitate effective coupling Ca^2+^ entry to activation of intracellular Ca^2+^ release.

### 3.3. The L-Type Ca^2+^ Channel and the Inositol 1,4,5-Trisphosphate Receptor Are Implicated in Ca^2+^ Release in CPCs in Response to Electrical Stimulation

**Figure 4:**
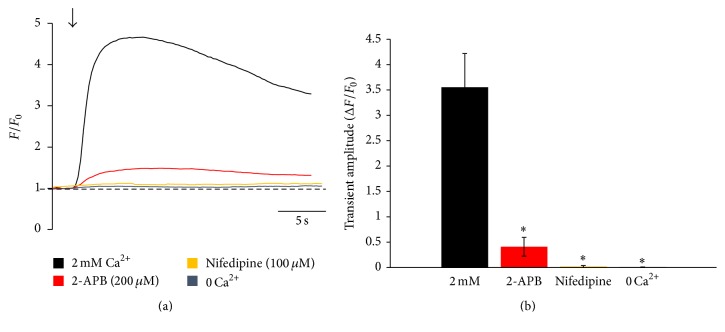
Cycling of Ca^2+^ in CPCs in response to electrical stimulation is dependent on the LTCC, IP_3_R, and external Ca^2+^. (a) Representative Ca^2+^ oscillations recorded from fluo-4/AM-loaded CPCs at 0.5 Hz electrical stimulation in 2 mM Ca^2+^ Tyrode's solution, pretreated with nifedipine or 2-APB to inhibit the LTCC or IP_3_R, respectively, or in 0 Ca^2+^ Tyrode's solution. Traces have been aligned in time based on when electrical stimulation was applied for comparison. Arrow indicates the start of stimulation. (b) Summary bar graph of Ca^2+^ oscillation amplitudes under the various recording conditions in (a). Data represent mean ± SD. ^*∗*^ *p* < 0.05; Student's *t*-test. *n* = 16 cells from 4 different pools of cells for all points.

In order to determine the proteins involved in the activation of Ca^2+^ release in CPCs, we used specific inhibitors of key Ca^2+^ handling proteins prior to electrical stimulation and also performed immunocytochemistry to confirm the expression of these proteins. Due to the fact that these cells readily responded to electrical stimulation, the voltage-dependent L-type Ca^2+^ channel (LTCC) was our first candidate for the protein mediating the response of CPCs to electrical stimulation. Pretreatment of cells for 5 min with the LTCC inhibitor nifedipine nearly abolished electrically induced increases in cytosolic Ca^2+^ in all cells recorded, suggesting that electrically induced Ca^2+^ oscillations in CPCs are dependent on the activity of the voltage-dependent LTCC (Figure 4). In adult cardiac myocytes, entry of Ca^2+^ into the cytosol through the LTCC in response to electrical stimulation initiates massive Ca^2+^ release from sarcoplasmic reticulum Ca^2+^ store through the ryanodine receptor (RyR). To determine whether the same process was present in CPCs, we first tested the necessity of extracellular Ca^2+^ for electrically induced Ca^2+^ oscillations in CPCs. Bathing the cells for 5 min prior to recording in an external solution without Ca^2+^ (0 Ca^2+^) prevented electrically induced Ca^2+^ release in CPCs (Figure 4). Pretreatment of the cells for 5 min with tetracaine, a RyR inhibitor, did not prevent electrically induced Ca^2+^ release in our CPCs (data not shown); however, pretreatment for 5 min with 2-aminoethoxydiphenyl borate (2-APB), an inositol 1,4,5-trisphosphate receptor (IP_3_R) inhibitor, significantly decreased the amplitude of electrically induced Ca^2+^ release (Figure 4). The IP_3_R is another intracellular Ca^2+^ release channel found in adult cardiac myocytes and has also been shown to mediate spontaneous Ca^2+^ release in multiple types of stem cells including CPCs [32, 35], and 2-APB has been shown to be an inhibitor of IP_3_R function, albeit a nonspecific one.

**Figure 5:**
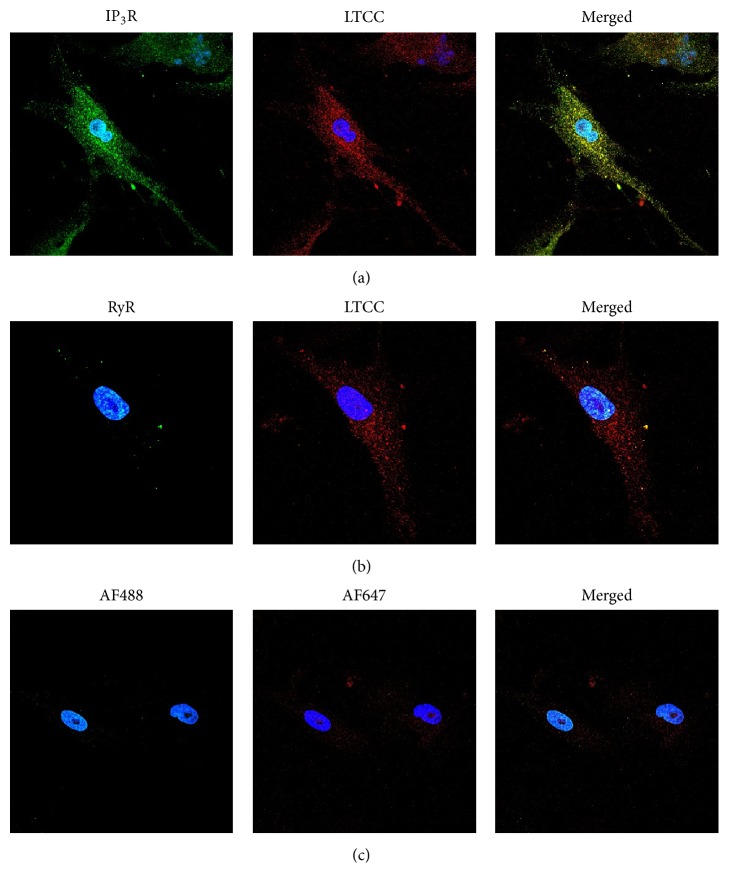
Immunostaining of CPCs shows expression and localization of the LTCC and IP_3_R. CPCs were fixed, permeabilized, and incubated with primary antibodies against the L-type Ca^2+^ channel (LTCC, Cav2.1 subunit), type 2 inositol 1,4,5-trisphosphate receptor (IP_3_R), or type 2 ryanodine receptor (RyR). Cells were then incubated with either Alexa Fluor 488 (IP_3_R and RyR; green) or Alexa Fluor 647 (LTCC; red). Nuclei are stained with DAPI (blue). Expression of the IP_3_R and LTCC is shown in (a), while expression of the LTCC and the RyR is shown in (b). Cells were also incubated with only secondary antibodies to determine nonspecific binding in (c). Images are representative of 3 replicates.

Immunocytochemistry confirmed the presence of the LTCC and the IP_3_R in CPCs, while no RyR was detected consistent with our functional studies (Figure 5). In summary, these data suggest that the LTCC and the IP_3_R are key proteins in electrically induced Ca^2+^ oscillations from human CPCs and represent a previously undescribed pathway for the activation of Ca^2+^ signaling in these cells; however, more experiments are necessary to confirm these results in human CPCs.

### 3.4. Long-Term Culture Stimulation Induces CPC Alignment

**Figure 6:**
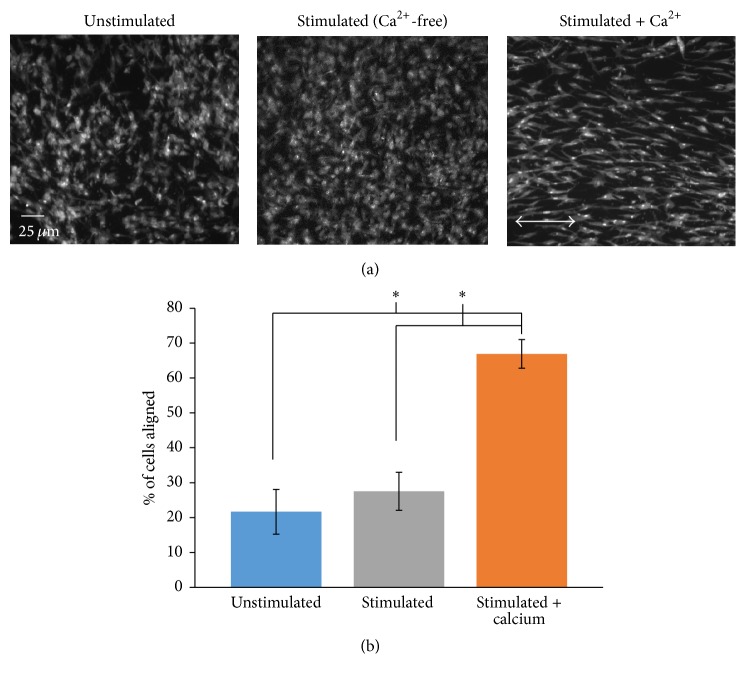
Electrically induced alignment of CPCs. CPCs were plated in stimulation media and subjected to electrical stimulation in culture for 72 hrs (stimulated + Ca^2+^). As controls, CPCs were plated in stimulation media and cultured for 72 hrs without electrical stimulation (unstimulated) or in Ca^2+^-free media and subjected to electrical stimulation (stimulated). All groups were fixed, permeabilized, and incubated with fluorescein-5-maleimide. Representative images of cells from the three groups are shown in (a). Summary bar graph of cellular alignment for the three culture conditions is shown in (b). Arrow in (a) (stimulated + Ca^2+^) is the direction of the electrical field. Data in (b) represent mean ± SD. ^*∗*^ *p* < 0.05; Student's *t*-test; *n* = 8 replicates.

Anisotropy of cardiac tissue is a key characteristic of the structure and function of the heart that facilitates electrical and mechanical activation of the myocardium. Alignment of cells organizes them into a tissue-like structure and has been shown to improve the differentiation of stem cells [36]. Therefore, we sought to determine whether long-term culture stimulation would induce this phenotypic change in CPCs. CPCs were cultured in stimulation media and subjected to 72 hrs of electrical stimulation. CPCs were also plated in stimulation media and cultured for 72 hrs without electrical stimulation to serve as an unstimulated control or stimulated in Ca^2+^-free media to serve as a stimulation control. Cells were fixed, permeabilized, incubated with 10 *μ*g/mL fluorescein-5-maleimide, and imaged. Representative images of unstimulated and stimulated CPCs are shown in Figure 6(a) and a summary graph of the quantified alignment scores is shown in Figure 6(b). Nearly 50% increase in the percent of cells aligned parallel to the electrical field was seen after 72 hrs of electrical stimulation compared to unstimulated cells or compared to stimulated cells in Ca^2+^-free media. These data illustrate an important phenotypic change induced in CPCs with chronic electrical stimulation and also indicated that this change is dependent on the presence of extracellular Ca^2+^. Our results showing that long-term culture stimulation induces Ca^2+^-dependent anisotropy in CPCs are important for the tissue architecture necessary for proper alignment of the cells in a tissue-like structure to prevent electrical conduction abnormalities, impaired pump function, or arrhythmias upon CPC implantation.

## 4. Conclusions

With Ca^2+^ handling linked, directly or indirectly, to almost all properties of cardiomyocytes including excitation-contraction coupling and excitation-transcription coupling, a solid understanding of this process in CPCs is crucial for fully realizing the cardiac therapeutic potential of these cells given recent studies that have shown that these cells do not significantly contribute to cardiac myocyte replenishment* in vivo* [4–6]. In this study, we have identified a novel property of human c-kit^+^ cardiac progenitor cells to respond to electrical stimulation by initiating intracellular Ca^2+^ oscillations. CPCs were isolated from human pediatric patients and pooled from multiple donors to diminish the effects of patient variability in our experiments. The use of human CPCs gives relevance to our findings for translation into a novel therapeutic strategy for clinical use, and our ability to perform live cell recordings of cytosolic Ca^2+^ in electrically stimulated CPCs represents a novel approach to understanding the physiology of these cells. As we have shown in this work, pediatric CPCs possess a broad spectrum of functional molecular elements of Ca^2+^ signaling. Ca^2+^-dependent regulatory mechanisms can be supposed to influence their differentiation potential, and we speculate that electrical stimulation activates a Ca^2+^-dependent signaling pathway in which the increased intracellular Ca^2+^ activates transcription of cardiospecific genes. In summary, we have characterized the electrically induced Ca^2+^ signal in CPCs by measuring the amplitude of cytosolic and nuclear Ca^2+^ oscillations elicited by electrical stimulation, identified the mechanism by which Ca^2+^ oscillations initiate and propagate throughout the cells, and also determined the key proteins involved in this process. Since our experiments were done in human pediatric CPCs, our results may be specific to that population of cells, and further experiments will be necessary to determine whether our results are species- or age-dependent [37]. To our knowledge, this is the first study to have reported the functional effect of acute* ex vivo* electrical stimulation on human CPCs. This study furthers our understanding of CPC physiology and may be used to direct future therapeutic strategies with these cells.

## Abbreviations

CPC: Cardiac progenitor cell
Ca^2+^: Calcium
LTCC: L-type Ca^2+^ channel
RyR: Ryanodine receptor
IP_3_R: Inositol 1,4,5-trisphosphate receptor.
